# Feasibility and Safety of Repeated Carbon Ion Radiotherapy for Locally Advanced Unresectable Pancreatic Cancer

**DOI:** 10.3390/cancers13040665

**Published:** 2021-02-07

**Authors:** Masahiko Okamoto, Shintaro Shiba, Shohei Okazaki, Yuhei Miyasaka, Kei Shibuya, Hiroki Kiyohara, Tatsuya Ohno

**Affiliations:** 1 Gunma University Heavy-ion Medical Center, Gunma University Graduate School of Medicine, Gunma 371-0811, Japan; shiba4885@yahoo.co.jp (S.S.); o_syohei_1015@yahoo.co.jp (S.O.); y.miyasaka@gunma-u.ac.jp (Y.M.); shibukei@gunma-u.ac.jp (K.S.); tohno@gunma-u.ac.jp (T.O.); 2 Japanese Red Cross Maebashi Hospital, Gunma 371-0811, Japan; hiroki.kiyohara@maebashi.jrc.or.jp

**Keywords:** carbon ion radiotherapy, pancreatic cancer, local recurrence, re-irradiation, locally advanced pancreatic cancer

## Abstract

**Simple Summary:**

Despite the effectiveness of carbon ion radiotherapy (CIRT) for locally advanced unresectable pancreatic cancer (URPC), isolated local recurrence after CIRT is a therapeutic challenge. Herein, we aimed to evaluate the feasibility and safety of a second course of CIRT for locally recurrent URPC in 10 patients. One patient developed grade 3 diarrhea immediately after the second CIRT; no other grade 3 or higher adverse events were induced by CIRT. The estimated 1-year overall survival rate and local control rate after the second CIRT were 48% and 67%, respectively. Repeated CIRT is feasible with acceptable toxicity for selected patients with locally advanced URPC after CIRT.

**Abstract:**

Purpose: The feasibility and safety of re-irradiation with carbon ion beams for locally recurrent unresectable pancreatic cancer (URPC) after carbon ion radiotherapy (CIRT) was evaluated. Methods: Medical records from patients with re-irradiated URPC who were treated with CIRT between November 2017 and February 2019 were reviewed. Inclusion criteria were (1) isolated local recurrence after CIRT, (2) URPC, and (3) tumor located at least 3 mm from the gastrointestinal tract. The first and second CIRT irradiation doses were 55.2 Gy (relative biological effectiveness) in 12 fractions. Results: Ten patients met the inclusion criteria. The median follow-up period was 25.5 months (range, 16.0–69.1) after the first CIRT and 8.9 months (range, 6.4–18.9) after the second CIRT. The median interval between the initial CIRT and the local recurrence was 15.8 months (range, 8.0–50.1). One patient developed grade 3 diarrhea immediately after the second CIRT; no other grade 3 or higher adverse events were attributed to CIRT. The estimated 1-year overall survival, local control, and progression-free survival rates after the second CIRT were 48%, 67%, and 34%, respectively. Conclusion: Repeated CIRT is feasible with acceptable toxicity for selected patients with locally advanced URPC after CIRT.

## 1. Introduction

Chemotherapy or chemoradiotherapy (CRT) is the standard of care for locally advanced unresectable pancreatic cancer (URPC). However, because the pancreas is surrounded by radiosensitive organs such as the stomach, duodenum, liver, kidneys, and intestines, delivering high dose X-ray irradiation is usually limited. Consequently, long-term local tumor control after irradiation for locally advanced URPC is insufficient. A meta-analysis comparing chemoradiotherapy with radiotherapy or chemotherapy alone from 15 randomized controlled trials (1128 patients) [1] showed that CRT resulted in superior 6- and 12-month survivals compared with radiation therapy or chemotherapy alone; however, the difference was not significant at 18 months. Even with high-tech radiotherapy, such as intensity-modulated radiation therapy, the median overall survival (OS) and the 2-year local control (LC) rate were 14–17 months and 39–42%, respectively [2,3].

Conversely, carbon ion radiotherapy (CIRT) has emerged as a promising therapy over the last two decades. The carbon ion (C-ion) beam has two advantages. First, because of the Bragg peak, the CIRT beam can be stopped at the end of the tumor, resulting in a decreased irradiated volume in healthy organs. Second, the relative biological effectiveness (RBE) of the CIRT high linear energy transfer is approximately three times higher than that of X-rays [4,5,6].

Several studies have focused on the efficacy and safety of CIRT for locally advanced pancreatic cancer [7,8]. In a multicenter retrospective study conducted in Japan [8], 72 patients were analyzed. In this study, CIRT resulted in a 2-year survival rate of 46%, median survival duration of 21.5 months, and cumulative local recurrence rate of 24% at 2 years after CIRT. Another single institutional study [7] showed that the 2-year LC was 63% based on the fluorodeoxyglucose (FDG)-positron emission tomography (PET) computed tomography (CT) criteria. According to these studies, 24–37% of patients with locally advanced URPC have a risk of local recurrence within 2 years after definitive CIRT. Patients without distant metastases at the time of local recurrence are suitable for local salvage treatment; however, salvage surgery is difficult in most cases when the original tumor is diagnosed to be unresectable. Re-irradiation with CIRT is a challenging approach owing to its biophysical advantage compared with X-ray irradiation. However, very limited data are available on the efficacy and safety of re-irradiation with CIRT for locally advanced URPC. In the present study, we report the feasibility and safety of re-irradiation with carbon ion beams for locally recurrent URPC after CIRT.

## 2. Results

### 2.1. Patient Characteristics

From November 2017 to December 2019, 10 patients met the inclusion criteria and underwent re-irradiation with CIRT. The patient characteristics are summarized in Table 1. All patients were diagnosed with T4 URPC at both the initial and second CIRTs. The median time to local recurrence after the initial CIRT was 15.8 months (range, 8.0–50.1). All patients received adjuvant chemotherapy after the first CIRT. Recurrences in the pancreas outside the initial CIRT target volume occurred in 2 of the 10 patients. All patients received 55.2 Gy (RBE) in 12 fractions for both the initial and second CIRT. For the re-irradiation, all patients completed the planned CIRT without any interruption. For the second CIRT, the irradiation dose was reduced to 52.8 Gy (RBE) in one patient to meet the dose constraints of the gastrointestinal tract. Eight of 10 patients also received concurrent chemotherapy during the second CIRT.

**Figure 1:**
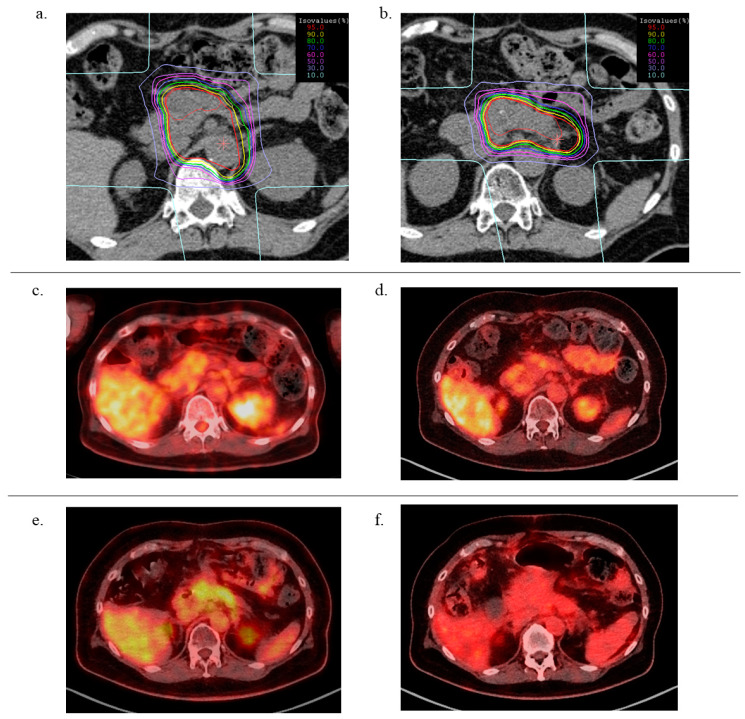
Typical case presentation: 55-year-old female patient with pancreatic body cancer. (**a**) Dose distribution of 1st CIRT, (**b**) dose distribution of 2nd CIRT, (**c**) fluorodeoxyglucose (FDG)-positron emission tomography (PET) computed tomography (FDG-PET/CT) image before 1st CIRT, (**d**): FDG-PET/CT image at 3 months after 1st CIRT, (**e**): FDG-PET/CT image at the recurrence after 1st CIRT, (**f**): FDG-PET/CT image at 3 months after 2nd CIRT.

A typical case (patient #8) is shown in Figure 1. The dose distribution of 1st CIRT indicates that the prophylactic lymph node lesions (such as the para-aortic area) were irradiated during the initial CIRT. Conversely, during the second CIRT, only the recurrent tumor was included in the clinical target volume (CTV). The FDG-PET/CT images showed that both the first and second CIRTs were effective in decreasing the standard uptake values of FDG 3 months after irradiation.

### 2.2. Survival and Local Control Rate

**Figure 2:**
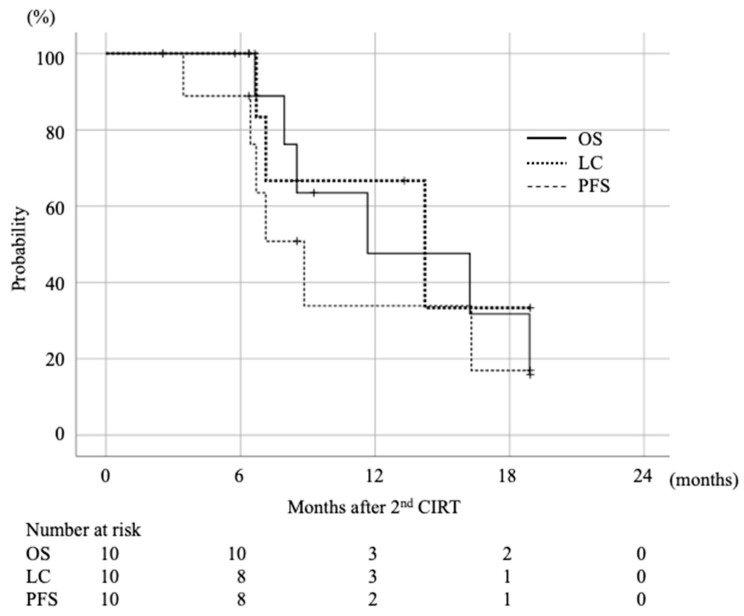
Survival rate after 2nd CIRT. The survival rates were calculated by using Kaplan–Meier method. OS; overall survival rate, LC; local control rate, PFS; progression free survival rate.

The median follow-up periods after the first and second CIRTs were 25.5 (range, 16.0–69.1) months and 8.9 (range, 6.4–18.9) months, respectively. The 1-year OS, LC, and progression-free survival (PFS) rates after the second CIRT were 48%, 67%, and 34%, respectively (Figure 2). The median survival duration and the 3- and 5-year survival rates after the initial CIRT were 37.0 (range 16.0–69.1) months, 53%, and 18%, respectively. Six patients relapsed after the second CIRT, with three local recurrences and three distant metastases at the second initial recurrent site. Of the three local recurrences, two were tumors that had been irradiated twice and one was a recurrence from a site that was irradiated only in the second CIRT. At the last follow-up, four patients had distant metastases, including peritoneal dissemination in two patients, a liver metastasis in one patient, and a lung metastasis in one patient. The univariate analysis with the endpoint of local recurrence is summarized in Table 2. Dose coverage (V95) for the gross tumor volume (GTV) was the only significantly different risk factor, with better LC in sufficiently irradiated cases with V95 of >90%.

### 2.3. Toxicities

Adverse events that occurred after the second CIRT are summarized in Table 3. The most frequent grade 2 or higher adverse event was cholangitis due to bile duct obstruction. In all periods, only one patient developed grade 3 diarrhea, which occurred immediately after CIRT (patient #9). The medical oncologist diagnosed diarrhea to be caused most likely owing to concurrent chemotherapy and the patient recovered. No other grade 3 or worse acute and late non-hematological toxicities were observed. Moreover, there were no treatment-related deaths.

### 2.4. DVH Comparison

Dose volume histragam (DVH) parameters between the first and second CIRTs are summarized in Table 4. Although the GTVs were the same between the first and second CIRTs, the CTV of the second CIRT was significantly smaller owing to the treatment policy for CIRT. Regarding the organs at risk (OAR), the second treatment resulted in significantly smaller irradiation volumes towards the stomach and intestine. In terms of GTV irradiation doses, approximately 97% of the GTV volume was covered by 90% of the prescribed dose for both the first and second CIRTs and 92–93% of the GTV was covered at 95% of the prescribed dose. Both treatments resulted in sufficient radiation to cover the GTV with no significant differences.

## 3. Discussion

The management of isolated local recurrences of URPC after definitive radiation therapy is challenging. Surgical resection is the most promising approach; however, it is often not possible in unresectable tumors or when prior radiotherapy causes fibrosis development in the area. Of note, pancreatic cancer is a systemic disease. However, when the recurrent tumor is truly localized and potentially curable but difficult to resect, there are currently no curative treatment alternatives. Our findings showed that re-irradiation with carbon ion beams is feasible with acceptable toxicities for selected patients with locally advanced URPC.

When considering re-irradiation using carbon ions, the development of severe adverse events is the most important concern. In this study, no grade 3 or higher late adverse events developed. Conversely, one grade 3 acute adverse event (diarrhea) developed in this study and the concurrent chemotherapy may have contributed or caused this adverse event. According to previous studies, the incidence of grade 3 or higher non-hematological toxicity caused by CIRT was approximately 4–10% [7,8]. Thus, the frequency of acute adverse events was comparable with that of the initial treatment. In the present study, we selected patients with tumors sized >3 mm from the external wall of the gastrointestinal tract and we prioritized OAR dose constraints over GTV coverage when planning treatments. Our results suggest that re-irradiation is safe with appropriate case selection.

The 1-year LC rate in the current study was 67%, which is slightly lower than the reported 84% LC rate for the first treatment [8]. This trend is similar to previous studies. Although the prescribed dose and DVH parameters, such as GTV V90 and V95, did not differ between the first and second CIRT, the univariate analysis showed that the LC rate was worse in the lower V95 group. Providing sufficient tumor coverage within the high dose area of at least 95% of the prescribed dose is important in improving the LC rate. Despite selecting cases where the distance between the tumor and the gastrointestinal tract is at least 3 mm for re-irradiation, the spacer insertion surgery might be effective to ensure adequate GTV coverage.

Another possible reason for the decreased LC rate after the second CIRT is the acquisition of radioresistance. At present, there are very few basic studies on the acquisition of radioresistance to carbon ion beams after CIRT. One report demonstrated that repeated carbon ion irradiation induced moderate radioresistance to carbon ions in a mouse squamous cell carcinoma cell line [9]. However, in mouse models, repeated carbon ion irradiation did not induce radioresistance [10]. In in vivo models, many factors affect radioresistance, including changes in the microenvironment and cancer stem cells. Further basic research is needed to clarify these issues.

Recently, several reports described re-irradiation for pancreatic cancer using C-ion radiotherapy [11], stereotactic body radiotherapy (SBRT) [12,13,14,15], and proton therapy [16]. Re-irradiation with C-ion radiation in 21 patients at a single center resulted in an LC rate of 53.5% and a 1-year survival rate of 48.7% [11]. Only one patient developed grade 3 acute toxicity and no grade 3 or higher late toxicities were observed. These results are very similar to those obtained in the present study. However, we observed a slightly higher LC rate, which may be due to the higher median CIRT dose (55.2 vs. 52.8 Gy RBE) in our study. Re-irradiation using SBRT resulted in a median survival period ranging from 5.9 to 14 months and a 1-year LC rate ranging from 62 to 78%. In the four studies on SBRT, 114 patients were treated, and 59 (52%) patients had a recurrence after radical surgery and adjuvant or neoadjuvant radiation therapy. In the case of re-irradiation after radical resection, several factors influence the occurrence of radiological adverse effects, including resection of the duodenum. Thus, a simple comparison of adverse events between resected and unresected cases is not possible. In addition, three of the four SBRT studies used only Response Evaluation Criteria in Solid Tumors (RECIST) criteria and did not consider FDG-PET when determining the efficacy of treatment, which is different from that in the present study. Proton beam re-irradiation for locally recurrent pancreatic cancer resulted in a median OS of 16.7 months and locoregional PFS of 72% in 15 patients [16]. In the proton beam re-irradiation study, 13 of 15 patients had a recurrence after radical surgery following preoperative irradiation, and only two patients received re-irradiation to the pancreas. Evaluation of LC was determined by tumor growth using only CT. Shinoto et al. reported that the 2-year LC based on RECIST and PET/CT criteria were 82% and 63%, respectively [7]. Thus, differences in patient background and evaluation criteria for LC influenced clinical outcomes. Alternatively, the grade 3 or worse non-hematological toxicities ranged from 6 to 26% after re-irradiation with SBRT or proton. In our study, the frequency of non-hematological toxicities was lower, despite the use of concurrent chemotherapy. Nevertheless, a long-term observation for toxicity is necessary.

For recurrence after radiation therapy, chemotherapy should be considered a standard therapy. We routinely recommend adjuvant chemotherapy after CIRT. However, chemotherapy regimens currently available for pancreatic cancer are limited, and there is often no effective chemotherapy for recurrence. Furthermore, CIRT can be used concurrently with chemotherapy to add a local effect to the benefits of systemic chemotherapy.

In recent years, re-irradiation with CIRT for head and neck tumors [17] and lung tumors [18,19] after CIRT was investigated. The 2-year LC rates for the second CIRT were 40.5%, 54%, and 66.9% for head and neck tumors, lung tumors including metastatic tumors, and stage I non-small–cell lung cancer, respectively. Although the LC rates were lower after the second CIRT than those in naïve cases, re-irradiation using carbon ions was reasonable because the toxicity was tolerable and there were limited treatment options at the time of recurrence.

Our study has some limitations. First, the study included a small number of patients. Second, the follow-up periods were relatively short. Conversely, this analysis suggests that adverse events following re-irradiation are acceptable under appropriate patient selection; hence, we will accumulate more cases and follow-up for a longer period.

## 4. Materials and Methods

### 4.1. Patient Eligibility

We reviewed the medical records of prospectively registered re-irradiated pancreatic cancer patients treated with C-ion radiation therapy at Gunma University Heavy Ion Medical Center (GHMC) between November 2017 and February 2019. The eligibility criteria for the re-irradiation were as follows: (1) history of CIRT for locally advanced URPC with curative intent; (2) unresectable disease at the time of recurrence; (3) no distant metastasis except for para-aortic lymph nodes that could be included in the irradiation target volume; (4) tumor location at least 3 mm from the external wall of the gastrointestinal tract; (5) Eastern Cooperative Oncology Group performance status of 0 to 2; and (6) well-informed and consented to join this treatment. Patients were excluded if they had (1) active ulcer of the stomach and/or duodenum; (2) active infection site in the upper abdomen; and (3) metallic stent inserted to treat obstructive jaundice. The treatment protocol of the current study was reviewed and approved by the Gunma University Ethics Committee of Human Clinical Research, and all patients signed an informed consent form before the initiation of therapy.

### 4.2. Carbon Ion Radiotherapy

The heavy ion accelerator at GHMC generated C-ion beams and 290 MeV/u, 380 MeV/u, and 400 MeV/u beam energy levels were selected according to the depth of the tumor. We used the XiO-N system (version 4.47; collaborative product of Elekta AB, Stockholm, Sweden and Mitsubishi Electric, Tokyo, Japan) for treatment planning. This system incorporates a dosing engine for ion beam RT (K2dose). MIM Maestro (MIM Software, Cleveland, OH, USA) was used to evaluate and sum up the treatment plans. We calculated the clinical radiation dose, which was expressed as Gy (RBE), based on the physical dose multiplied by the RBE of the C-ion beams [20]. Before C-ion RT, patients were immobilized using tailor-made fixation cushions and thermoplastic shells to acquire treatment planning CT images; respiratory-gated and 4-dimensional CT images and dynamic contrasted CT images were then acquired. Patients received C-ion RT once daily, 4 days a week (Tuesday–Friday).

### 4.3. Treatment Planning

The treatment planning CT images were merged with the dynamic enhanced CT and magnetic resonance imaging and FDG-PET CT images to delineate the GTV. The CTV was defined as the GTV, including at least 5 mm margins in all directions; for initial CIRT, 1 cm margins in the long axis of the pancreas were added. The prophylactic lymph node and neuroplexus regions were included in the initial CIRT but not the second CIRT. The planning organ at risk volume (PRV) was obtained by adding a 2 mm margin to the gastrointestinal tract. Areas that overlapped with the PRV of gastrointestinal tract were excluded from the CTV. The planning target volume (PTV) included the CTV with a 3 mm margin for possible positioning errors. When the PTV overlapped with an organ at risk, the margin was accordingly reduced. The maximal irradiated doses of the stomach and duodenum were restricted to <45 Gy (RBE). The stomach and duodenum volumes that were irradiated with >30 Gy (RBE) were kept under 10 cm^3^. The maximum irradiation dose to the spinal cord was restricted to <30 Gy (RBE). The dose constraints were generally based on the same criteria for the first and second treatments, but more restrictive dose constraints were used for the second treatment for patients in whom the gastrointestinal tract was close, based on the decision of the radiation oncologist. Based on the anatomy of the pancreas, CT for treatment planning for the first and second CIRTs is rigidly matched on the radiation treatment planning system to confirm the dose distribution for the surrounding risk organs. Then, the dose distribution of the second CIRT was optimized such that the 70% isodose lines of the first and second treatments did not overlap on the structures of the gastrointestinal tract. Prescribed doses were 55.2 Gy (RBE) in 12 fractions for standard cases. If the dose constraints for the OAR did not meet the criteria, the prescribed dose was reduced to 52.8 Gy (RBE) in 12 fractions for the second CIRT.

### 4.4. Evaluation and Follow-Up

Acute and late toxicities were graded according to the National Cancer Institute Common Toxicity Criteria (version 4.0). The highest grade within 3 months and after 3 months was evaluated as acute and late toxicity, respectively. Tumor response was evaluated by comparing pre- and post-treatment CT scans and FDG-PET scans and the RECIST was used for evaluation. When increased FDG accumulation was detected in the tumor evaluated using RECIST, the tumor was determined to be a progressive disease. Residual FDG accumulation 6 months after treatment was considered a local recurrence. Follow-ups were performed at least every 6 months. CT and/or FDG-PET scans, tumor markers, physical examination, and interviews were performed at each visit.

### 4.5. Statistical Analysis

The LC, PFS, and OS rates were calculated using the Kaplan–Meier method. Local recurrence was defined in terms of lesions occurring in the PTV according to CT or FDG-PET scans. LC was defined by the absence of local recurrence, as indicated by no evidence of an increase in tumor size by >20% on CT and no accumulation by FDG-PET at >6 months after treatment. Univariate analysis was performed, and the log-rank test was used to compare subgroups. A *p*-value of <0.05 was considered statistically significant. All statistical analyses were conducted using IBM SPSS Statistics for Mac, version 26.0 (SPSS, Armonk, NY, USA).

## 5. Conclusions

Although the observation period and the number of cases are not yet sufficient, carbon ion re-irradiation for local recurrence after CIRT for locally advanced pancreatic cancer had acceptable adverse events and a favorable LC rate. It is suggested that re-irradiation with carbon ion is an effective treatment option for patients with local recurrence and no obvious distant metastases.

## Figures and Tables

**Table 1:** Patients and CIRT characteristics.

pt.#	Age	Sex	1st CIRT					2nd CIRT				
			TNM (UICC 7th)	RT Dose (Gy(RBE))	Tumor Location	Concurrent CT	Adjuvant CT	CIRT Interval (M)	Tumor Location	RT Dose (Gy(RBE))	Concurrent CT	Adjuvant CT
1	68	F	T4N0M0	55.2	Pb	GEM	GEM	25.3	in-field	52.8	GEM	GEM
2	63	F	T4N1M0	55.2	Pt	S-1	S-1	24.6	in-field	55.2	GEM	GEM
3	78	M	T4N0M0	55.2	Ph	S-1	GnP	12.7	out-field	55.2	S-1	S-1
4	49	M	T4N0M0	55.2	Pb	GEM	GEM	50.1	in-field	55.2	GEM	GEM
5	45	M	T4N0M0	55.2	Pb	S-1	S-1	8.0	in-field	55.2	S-1	S-1
6	51	M	T4N0M0	55.2	Pb	GEM	GEM	9.3	out-field	55.2	GEM	GnP
7	70	F	T4N0M0	55.2	Pb	S-1	S-1	11.0	in-field	55.2	S-1	S-1
8	55	F	T4N0M0	55.2	Pb	S-1	S-1	13.8	in-field	55.2	S-1	none
9	67	M	T4N0M0	55.2	Ph	GEM	GnP	26.0	in-field	55.2	none	S-1
10	78	M	T4N0M0	55.2	Pb	GEM	GEM	17.8	in-field	55.2	none	GEM

pt: patient, CIRT: carbon ion radiotherapy, RT: radiation therapy, CT: chemotherapy, M: months, RBE: relative biological effectiveness, Ph: pancreatic head, Pb: pancreatic body and tail, GEM: gemcitabine, GnP: gemcitabine with nab-pacritaxel.

**Table 2:** Univariate analysis for local recurrence after 2nd CIRT.

Factor	Category	Number	95% CI	*p*-Value
age	≥65	5	6.9–8.8	0.695
	<65	5	9.3–18.9	
duration for recurrence	≥1 year	7	11.0–21.0	0.224
	<1 year	3	3.1–17.8	
recurrence site	in field	8	9.5–18.9	0.450
	out of field	2	6.4–8.9	
GTV V95 (%)	≥90	7	13.3–19.8	0.018
	<90	3	6.5–7.3	
GTV V90 (%)	≥90	8	10.3–18.9	0.247
	<90	2	7.1–7.1	

GTV: gross tumor volume.

**Table 3:** Numbers of cases for each non-hematological adverse event after 2nd CIRT.

Adverse Event	Grade 0–1	Grade 2	Grade 3	Grade 4–5
upper GI	8	2	0	0
lower GI	7	2	1	0
biliary tract	4	6	0	0
portal vein	7	3	0	0
dermatitis	10	0	0	0
peripheral nerve	10	0	0	0
pain	10	0	0	0

**Table 4:** Comparison of the DVH parameters between 1st and 2nd CIRT.

DVH Parameters		Mean (SD)	*p*-Value
GTV volume (cc)	1st CIRT	21.9 (16.8)	0.47
	2nd CIRT	16.6 (15.7)	
CTV volume (cc)	1st CIRT	140.1 (37.6)	<0.001
	2nd CIRT	38.6 (22.9)	
GTV V90%dose (%)	1st CIRT	96.8 (3.5)	0.97
	2nd CIRT	96.7 (3.6)	
GTV V95%dose (%)	1st CIRT	93.4 (6.3)	0.79
	2nd CIRT	92.6 (7.1)	
Stomach—D2cc (Gy(RBE))	1st CIRT	32.1 (6.5)	0.004
	2nd CIRT	17.5 (11.5)	
Stomach—V30Gy (cc)	1st CIRT	4.3 (3.0)	0.004
	2nd CIRT	0.7 (0.9)	
Duodenum—D2cc (Gy(RBE))	1st CIRT	29.5 (7.0)	0.21
	2nd CIRT	24.9 (8.4)	
Duodenum—V30Gy (cc)	1st CIRT	3.4 (2.8)	0.47
	2nd CIRT	2.4 (3.0)	
Intestine—D2cc (Gy(RBE))	1st CIRT	32.1 (7.2)	0.02
	2nd CIRT	22.1 (10.2)	
Intestine—V30Gy (cc)	1st CIRT	6.4 (3.7)	0.004
	2nd CIRT	1.7 (2.7)	

DVH: dose volume histogram, CIRT: carbon ion radiotherapy, SD: standard deviation.

## Data Availability

The data presented in this study are available on request from the corresponding author. The data are not publicly available due to privacy and ethical restriction.
